# Broadening risk factor or disease definition as a driver for overdiagnosis: A narrative review

**DOI:** 10.1111/joim.13465

**Published:** 2022-03-20

**Authors:** João Pedro Bandovas, Beatriz Leal, Catarina Reis‐de‐Carvalho, David Cordeiro Sousa, João Cruz Araújo, Pedro Peixoto, Susana Oliveira Henriques, António Vaz Carneiro, the Choosing Wisely Working Group of the European Federation of Internal Medicine

**Affiliations:** ^1^ Department of General Surgery Centro Hospitalar Universitário de Lisboa Central Lisboa Portugal; ^2^ Department of Anesthestics Instituto Português de Oncologia de Lisboa Francisco Gentil Lisboa Portugal; ^3^ Department of Obstetrics, Gynecology and Reproductive Medicine Centro Hospitalar Universitário Lisboa Norte Lisboa Portugal; ^4^ Vision Sciences Study Center Faculdade de Medicina Universidade de Lisboa Lisboa Portugal; ^5^ Vitreoretinal Unit Royal Victorian Eye and Ear Hospital Melbourne Australia; ^6^ Family Medicine Department Unidade de Saúde Familiar Gualtar Braga Portugal; ^7^ Department of Family Medicine Unidade de Saúde Familiar do Mar Póvoa de Varzim Portugal; ^8^ Central Library Faculdade de Medicina Universidade de Lisboa Lisboa Portugal; ^9^ Institute for Evidence Based Healthcare Faculdade de Medicina Universidade de Lisboa Lisboa Portugal

**Keywords:** changing risk factor/disease definitions, disease definition, low‐value care, overdiagnosis, overtreatment, resource overuse

## Abstract

Medical overuse—defined as the provision of health services for which potential harms exceed potential benefits—constitutes a paradigm of low‐value care and is seen as a threat to the quality of care. Value in healthcare implies a precise definition of disease. However, defining a disease may not be straightforward since clinical data do not show discrete boundaries, calling for some clinical judgment. And, if in time a redefinition of disease is needed, it is important to recognize that it can induce overdiagnosis, the identification of medical conditions that would, otherwise, never cause any significant symptoms or lead to clinical harm. A classic example is the impact of recommendations from professional societies in the late 1990s, lowering the threshold for abnormal total cholesterol from 240 mg/dl to 200 mg/dl. Due to these changes in risk factor definition, literally overnight there were 42 million new cases eligible for treatment in the United States. The same happened with hypertension—using either the 2019 NICE guidelines or the 2018 ESC/ECC guidelines criteria for arterial hypertension, the proportion of people overdiagnosed with hypertension was calculated to be between 14% and 33%. In this review, we will start by discussing resource overuse. We then present the basis for disease definition and its conceptual problems. Finally, we will discuss the impact of changing risk factor/disease definitions in the prevalence of disease and its consequences in overdiagnosis and overtreatment (a problem particularly relevant when definitions are widened to include earlier or milder disease).

## Graphical abstract

Content List – This is an article from the symposium: “When is too much too much and too little too little: Scientific evidence of limited workup”.

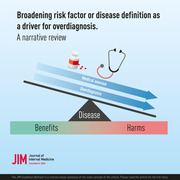

## Introduction

Autism is a neurodevelopmental disorder whose clinical picture includes persistent deficits in social interaction and communication, as well as repetitive patterns of behaviour. In its most severe form (level 3 out of 3 levels), it is a very serious condition, requiring full support from a multidisciplinary team according to the child's age and specific needs [1]. The putative reclaimed causes have included, among others, parents' shortcomings, preventive vaccines, heavy metal contamination, herbicides, electromagnetic radiation, gluten and casein. None of these putative causal relationships has ever been proven to be true, so the aetiology remains elusive.

In the past two decades, the proportion of child population the Centre for Disease Control has identified with autism has gone from 6.7 cases per 1000 in 2000 to 18.5 per 1000 in 2016 [2]—a 176% increase in prevalence. Table 1 shows the evolution of the classification by the Autism and Developmental Disabilities Monitoring (ADDM) Network, a group of programs funded by CDC to estimate the number of children with autism spectrum disorder and other developmental disabilities living in different areas of the United States. This looks like a serious mental/neurological health issue, needing a solid approach for diagnosis and treatment. But is it?

**Table 1:** Identified prevalence of autism spectrum disorder (Autism and Developmental Disabilities Monitoring [ADDM] criteria)

Surveillance year	Combined prevalence per 1000 children (range across ADDM sites)	Absolute risk (e.g., 1 in X children)
2000	6.7 (4.5–9.9)	1 in 150
2004	8.0 (4.6–9.8)	1 in 125
2008	11.3 (4.8–21.2)	1 in 88
2012	14.5 (8.2–24.6)	1 in 69
2016	18.5 (18.0–19.1)	1 in 54

The diagnosis of autism as a disease is mostly based on a set of criteria detailed in two main sources—the Diagnostic and Statistical Manual of Mental Disorders (DSM) and the International Disease Classification (ICD). Both rely on questionnaires addressing persistent deficits in social communication and interaction and restricted and repetitive types of behaviour or activities, with impact in daily life on different age groups.

The DSM definitions of autism have changed significantly over the years, and these changes are as follows [3]:

- The 1952 edition of DSM (DSM‐II) defined autism as a psychiatric condition—a form of childhood schizophrenia marked by a detachment from reality. During the 1950s and 1960s, autism was thought to be rooted in cold and detached mothers, called ‘refrigerator mothers’. This concept was disproved in the 1960s and 1970s, based on a growing body of research showing that autism has biological causes and is related to brain development.
- The DSM‐III (1980) defined autism as a ‘pervasive developmental disorder’, rooted in brain development, with the following diagnostic criteria (met before 30 months of age): (i) lack of responsiveness to other people, (ii) gross deficits in language development and (iii) bizarre responses to the environment.
- The 1987 revised version of the DSM‐III broadened the diagnosis to include milder cases through a list of 16 diagnostic criteria (patients had to meet eight of them) and allowed children over 30 months to be diagnosed with the disease. In this third edition, autism was subdivided into ‘Autism’ and ‘Pervasive Developmental Disorder—Not Otherwise Specified’ (PDD‐NOS), allowing clinicians to include children without the full criteria for autism, but still requiring developmental or behavioural support.
- The 1994 DSM‐IV edition introduced the word ‘spectrum’. It included four new different conditions: ‘Asperger's syndrome’, ‘Pervasive Developmental Disorder—Not Otherwise Specified’, ‘Childhood Disintegrative Disorder (CDD)’ (at the lowest end of the spectrum) and ‘Rett Syndrome’ (mainly affecting girls).
- The most recent edition of DSM (DSM‐5) was published in 2013. In this edition, ‘Childhood Disintegrative Disorder’ and ‘Rett Syndrome’ were dropped. There is now an ‘Autistic Spectrum Disorder’, lumping three diagnoses together, with no clear distinctions between them and characterized by two groups of features—'Persistent Impairment In Reciprocal Social Communication and Social Interaction’ and ‘Restricted, Repetitive Patterns of behaviour’, both present in early childhood.

Looking at this context, the explanation for the increase in the prevalence of autism may be simply since its definitions repeatedly changed over the years, from no diagnostic set of criteria in the 1950s to five diagnostic standards in the 1990s, and from these to a very broad definition nowadays. By enlarging the diagnostic definitions and in the absence of any typical and reproducible changes in analytic or imaging diagnostic data, many more people now fall into the categories of autism.

Suppose one prefers to resort to the 10th edition of the International Classification of Diseases (released in the 1990s). In that case, the problem remains—this manual groups autism, Asperger syndrome, Rett syndrome, CDD and PDD‐NOS together in a single ‘Pervasive Developmental Disorders’ (much like the DSM‐IV did).

As said, the expansion of the criteria for diagnosing autism—by broadening the diagnostic features, as well as including more subpopulations at risk (young adults, very young children, etc.)—increases artificially the prevalence of disease and, therefore, induces overtreatment of patients at very low risk of having the disease. Another, subtler cause for overdiagnosis may be that, although not much has changed in terms of the distribution in the population of the psychological traits that we now associate with ‘autism’, healthcare professionals changed what they were measuring, looking out more intensely for autism. This practice suffers from ascertainment bias—defined as a systematic distortion in measuring the true frequency of a phenomenon due to how the data are collected—and is hard to detect and quantify.

In this review article, we discuss resource overuse in modern health systems and its causes and consequences for the quality of care. We then present the criteria for disease definition and the difficulties that one can encounter sometimes for defining a specific pathologic entity. Next, we discuss the impact of changing risk factors and disease definitions, using some well‐performed studies. The conclusion will address some solutions to address resource overuse in modern practice.

## Resource overuse

Medical overuse constitutes an increasingly recognized problem in contemporary healthcare. It is defined as the use of unnecessary health services—either tests, procedures or treatments—that do not provide clear benefits for patients' health and can even cause harm [4]. Through unnecessary screening and diagnostic testing, overuse may lead to overdiagnosis, which comprehends the identification of medical conditions that would, otherwise, never cause any significant symptoms or lead to clinical harm [4]. On the other hand, overuse can also be a consequence of overdiagnosis itself, which can lead to labelling and unnecessary follow‐up testing and treatments. Therefore, it is recognized as a relevant threat to value, safety and quality of care in contemporary healthcare systems.

The negative consequences to patients of overuse fall in a broad range of distinct domains, such as psychological, physical, social, financial, treatment burden and dissatisfaction with healthcare services [5]. Psychological harm correlates with the negative emotional impact caused by the stress and uncertainty that treatments or tests may trigger and the impact of being labelled ‘ill’ due to an unnecessary procedure. Physical consequences include the risk of complications from medical procedures and adverse drug reactions, leading to disability or death. From a social perspective, a misdiagnosed medical condition can lead to psychological harm, potentially disrupting relationships and social stigma. Financial consequences may be direct, related to monetary costs of medical interventions and indirect, because of work absence, loss of productivity and eventual job loss. Medical overuse also leads to more time spent with unnecessary medical care, resulting in an increased workload for patients because of the treatment burden. All these factors contribute, in a broader perspective, to dissatisfaction and distrust with the healthcare system. For example, an unnecessary colonoscopy may lead, on a short‐term basis, to gastrointestinal symptoms related to bowel preparation (physical domain), which in turn may trigger anxiety (psychological domain). The risk of certain complications during the procedure, such as bowel perforation (a rare event), may lead to long‐term morbidity and, ultimately, dissatisfaction and tension with healthcare services and physicians, loss of productivity (financial domain) and increased medical bills. This situation can create a feedback loop in the overuse cascade, where the complications caused by the unnecessary procedure lead to additional treatments, which in turn contribute to potential additional harm.

Healthcare systems are also subject to harm because of medical overuse, which may cause a significant financial impact due to increased expenditure from the rising volume of medical services [6]. In Australia, overuse has been identified as a bigger cause of health cost increases than population growth or ageing. In the United States, a conservative analysis estimated that, in 2013, a minimum of $270 billion was spent on overuse, even though a significant part of the population still lacks access to healthcare services [6]. Based on these data, it is possible to conclude that overuse poses a threat to healthcare systems' sustainability, negatively impacts resource distribution and ultimately leads to a decrease in quality of care.

Multiple factors are contributing to overdiagnosis and overuse [7]. Broadening disease definitions, as discussed below, leads to higher prevalence rates and may cause potential harm without changing outcomes. The increasing accessibility and use of advanced medical diagnostic equipment, including more sensitive screening tests, also plays an important role due to a rise in the incidence of incidental findings with low clinical significance, which may trigger a cascade of unnecessary tests and treatments [8].

Public health screening programs represent a common prevention strategy that aims to identify specific diseases early in their development. However, the potential benefits may not outweigh the harms and associated costs. The fact that it drives to the identification of disease along its spectrum of severity, including very low risk and indolent conditions that might never become clinically significant during a lifetime, may result in a significant degree of overdiagnosis. Prostate cancer flagged by screening programmes provides an excellent example since an important proportion of patients are treated for indolent tumours with a low chance of causing clinical consequences if left alone. In turn, this overdiagnosis may lead to overtreatment with the potential for side effects, such as impotence and incontinence [8]. Current medical practice in many countries, characterized by intolerance to uncertainty, the need to label every problem with a diagnosis, pervasive risk aversion, fear of malpractice and litigation are also important drivers of overdiagnosis and overuse. The behaviour adopted by healthcare professionals can also be influenced by the expectations and increased demand for medical care expressed by patients. On the other hand, financial incentives from ordering tests and performing medical and surgical procedures may result in increasing demand [9].

Healthcare professionals are a major driver for resource overuse. Physicians' lack of knowledge concerning diagnostic methodology, the natural course of the disease and appropriate complex therapeutic pathways as well as difficulty in interpreting information—either from physical examination, diagnostic tests or scientific evidence—can contribute to overuse as a compensatory strategy. Limitations related to existing evidence also pose a risk of overdiagnosis and overuse—for example, clinical trials may fail to reveal and discuss the heterogeneity of treatments related to patient differences in disease severity, risk of adverse effects and responsiveness to treatment. However, from a physicians' perspective, the main reason frequently quoted as a driver for overuse was defensive medicine, related to fear of malpractice [7].

Medical consumerism may be a relevant driver for resource overuse. By requesting more and more tests in their clinical encounters, consumers believe that this approach constitutes a high‐quality type of care, carrying no harm. A retrospective study on the preventive use of whole‐body computed tomography (CT) screening for cancer in 1192 patients—76% of which were self‐referrals—found 86% of the participants to have at least one abnormal finding. Overall, there were 2.8 findings per patient, and 37% of these received at least one recommendation for further evaluation [10].

Multiple examples are showing that medical overuse can lead to a futile cascade of events, exams and treatments. A common example is the prescription of imaging in the workup of low back pain (LBP): in an ambulatory setting, only 5%–10% of these patients present with suspicious LBP (neurologic findings, for example) where additional diagnostic imaging is necessary. Despite this, around one quarter of patients are imaged, as are around one third in emergency departments. It has been determined that the rate of complex imaging per patient increased around 50% between 1995 and 2015, raising concerns about overuse and its consequences [11, 12]. Given the progression of knowledge about imaging in LBP, a decrease in imaging prescription rates should be expected. However, recent systematic reviews demonstrate the opposite, reporting a significant increase over the past 20 years [11].

The approach described above poses a risk of harm, including increased exposure to radiation and the possibility of leading to incidental findings—named ‘incidentalomas’—especially with techniques that provide more detailed images, such as computerized tomography scans and magnetic resonance imaging [13]. Incidentalomas constitute a potential source of anxiety and concern for patients, especially when the physician does not correctly explain its meaning or, as it so frequently happens, promotes further investigations, specialist referrals and more intensive and often unnecessary medical or surgical procedures. Disc abnormalities, such as degenerative disc disease or bulging disc, are commonly described in imaging exams, although they may not be the cause of pain since they are also seen in up to 97% of asymptomatic patients [14]. To complicate this problem, there is a very marked variation in the interpretation of imaging in LBP, even from the same radiologist [15].

**Fig. 1:**
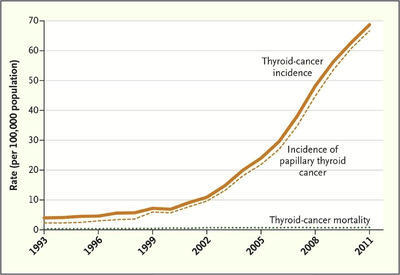
Thyroid‐cancer incidence and related mortality in South Korea, 1993–2011 [12] (in process of obtaining a license for republication permissions, through the Copyright Clearance Center [CCC]).

A paradigmatic example of overdiagnosis occurred in South Korea between 1999 and 2008, with a steep increase in the diagnosis of thyroid cancer, because of a government‐funded national cancer screening program, which led to the widespread use of thyroid ultrasound in asymptomatic people [16, 17]. Consequently, the incidence of papillary thyroid cancers increased 15 fold, whereas the mortality rate remained unchanged (Fig. 1) [6, 12]. In this context, it is estimated that 99.7%–99.9% of all thyroid cancers diagnosed in South Korea represent overdiagnosis, mostly of small papillary tumours [15]. Whereas in the past, most thyroid cancers were diagnosed in patients who presented with symptoms related to compression caused by nodules, visible neck masses, or through regular physical examinations, the advent of neck ultrasonography and ultrasound‐guided biopsy allowed detection and biopsy of nodules as small as 2 mm [15]. Wider and increased use of advanced and more sensible imaging technology for other indications, such as CT and magnetic resonance, has also contributed to the rise in incidence, allowing the incidental finding of even smaller nodules [18]. In fact, in the United States, approximately 16% of CT and magnetic resonance images show incidental thyroid nodules, of which around three quarters are <15 mm.

Considering that patients then submitted to unnecessary thyroidectomy face an 11% risk of hypoparathyroidism and a 2% risk of vocal cord paralysis, the described situations represent clear downstream harm of overdiagnosis and overtreatment [6]. Thyroid cancer screening benefits have never been duly documented, and the extent to which opportunistic screening is converting asymptomatic and healthy persons to cancer patients, without any known benefit to them, needs to be carefully examined [16].

Another example of low‐value care is the annual resting electrocardiogram (ECG), often prescribed in a primary care setting in low‐risk patients. A study found that asymptomatic people who had an ECG following an annual health examination were five times more likely to have additional cardiac tests, procedures or consultation with a specialist than those who did not, and with no benefits in combined rates of death, cardiac‐related hospitalizations and coronary revascularizations [19]. On the other hand, echocardiography has shown inconsistent results. It appears to be inappropriately underused for some clinical situations, for example, confirming a diagnosis of heart failure, and inappropriately overused for others, such as in the routine perioperative evaluation of ventricular function with no symptoms or signs of cardiovascular disease [20].

One can also find evidence of overutilization concerning inpatient physical therapy; a recent study found that hospital services at one academic medical center may have been overutilizing inpatient physical therapy consults. In a retrospective cohort of 3592 patients hospitalized for longer than 48 h, 38% of physical therapy consults were deemed potential overutilization based on Activity Measure–Post Acute Care (AM‐PAC) Inpatient Mobility Short Form scores [21].

Overuse is a common worldwide problem in healthcare, and patients should be aware of its consequences. But this may not be easy; in a recent randomized controlled trial (RCT), 775 individuals received brief written information about three low‐value care interventions—prostate cancer screening in men aged 50–69 years, osteoporosis screening in low‐risk women aged 50–64 years or colorectal cancer screening in men and women aged 76–85 years [22]. Despite receiving different formats of clinical information—narrative, quantitative, mixed words and numbers—the intervention was insufficient to change patients' intentions for screening. These results should lead us to question our approach to health literacy and develop alternate and additional interventions to sensitize people to reject low‐value screening.

What are the solutions for addressing resource overuse? Physicians' central role in key clinical decisions and resource allocation represents an opportunity for improvement. Although healthcare spending varies significantly among hospitals, a recent study concluded that this difference is even more pronounced across individual physicians, with no established correlation between higher spending and clinical benefits [23]. This raises questions about the importance of considering and planning tailored interventions to change practices according to specific circumstances.

Future strategies to reduce overuse should also focus on medical education. The analysis of differences between training and nontraining hospitals may provide important clues. A cross‐sectional study conducted in the United States, which involved patients hospitalized with cellulitis or bacterial pneumonia, concluded that the average number of laboratory tests prescribed per day and patient was higher in teaching hospitals when compared to nonteaching institutions [24]. This association remained significant after adjustments for illness severity and patient demographics. Although its primary objective did not include the analysis and comparison of clinical outcomes, hence not allowing for conclusions on resource overuse, the study showed evidence that residency training contexts are associated with a trend of increased laboratory testing. Several reasons leading up to the described situation were considered, such as lack of knowledge, lack of clinical experience and insecurity about testing criteria and, concerning resident physicians, the fact that supervising doctors may be more prone to complain about missing morning laboratory test results rather than unnecessary and excessive tests [24]. Another reason for this situation may represent an underestimation of careful history taking and physical examination, as well as an overestimation of laboratory tests independently on their specificity and accuracy.

It is crucial to conceptualize overuse as a general healthcare system problem. Given its broad negative consequences, every stakeholder from patients to physicians and policymakers should be engaged in reducing the use of services for which the potential for patient harm exceeds its benefits.

## Disease definition

What constitutes a disease is a fundamental question for modern medicine. For a patient to benefit from having a disease properly diagnosed, it must facilitate the understanding of his clinical picture, help establish a rigorous prognosis, or benefit the patient with a new or specific treatment.

Evidence about clinical signs and symptoms of the disease usually comes from clinical care research studies. A sample of patients believed to have the disorder of interest is selected, and the presence or absence of each finding in each patient is determined (sometimes with its qualitative features in time and clusters or patterns of findings). To analyze the validity of this approach, one must know how the diagnosis was verified, how similar the patients in the sample are in relation to all patients with the disease, how the collection of clinical findings was done and its characterization [25].

It is often difficult to accurately explain what disease is. A common definition—based on a constellation of signs and symptoms—must consider that these clinical signs and symptoms often do not have discrete boundaries but rather exist on a continuous scale [26]. For example, there is ongoing discussion regarding the increasing number of children diagnosed with attention deficit hyperactivity disorder, but how to reliably define a threshold between what is bad behaviour or a real condition [27, 28]? Another example is the criteria for female sexual dysfunction, motivated by the huge commercial success in treating erectile dysfunction in men [29, 30].

Doctors tend to interpret the disease as a fixed concept, even though the thresholds between what is normal and abnormal are poorly defined and based on limited scientific evidence. The way we make diagnoses and decide who to treat greatly contributes to this. For example, a set of inclusion and exclusion criteria for a given disease are previously defined in randomised controlled trials. The participants who fulfil this checklist are classified as patients, and those who do not fulfil it are potential healthy controls. However, a problem arises when this checklist changes and the results of the trials only apply to a small proportion of the real‐world population [31].

## Importance of disease definitions

As Doust et al. stated in their paper, ‘…Being diagnosed with a disease only benefits a patient if the diagnosis assists in understanding current symptoms or the risk of future clinically important events, or if the patient can benefit from a specific treatment. To appreciate potential harms and benefits of the change in definition, it is necessary to understand the natural history for those patients labelled by the new definition but not by the previous definition’ [32].

**Fig. 2:**
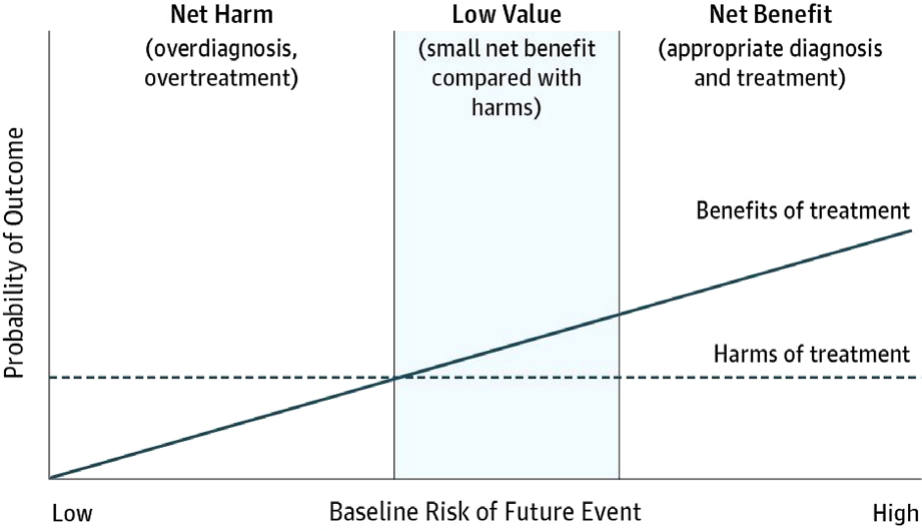
Baseline risk and efficacy of treatment [32] (in process of obtaining a license for republication permissions, through the Copyright Clearance Center [CCC]).

The changes in diagnostic criteria are sometimes necessary, for example, after the development of a more sensitive test and when there is new data on prognosis. Moreover, widening the diagnostic criteria of a disease may not always be harmful per se. However, multiple randomised studies that stratify patients by baseline risk have concluded that there are significant differences in the impact of treatment: patients with milder disease are less likely to benefit from intervention when compared with high‐risk patients (Fig. 2) [3]. Recently, the SPRINT trial was used to support the lower diagnostic threshold of hypertension in the 2017 definition. This study enrolled patients with systolic blood pressure of 130 mmHg or higher and a high increased baseline cardiovascular disease risk. As with other trials—including patients with a similar blood pressure level but a lower baseline cardiovascular risk—there was no benefit from treatment. On the other hand, this inclusion of patients with milder or earlier disease may bias the interpretation of the real treatment effect—that is, the ratio of well‐controlled patients by the total number of cases will appear to improve, even when there is no true effect [33]. Another common misbelief is that an earlier diagnosis will trigger positive lifestyle modifications. However, multiple trials disproved this idea, showing that even highly personalized risk information does not change health‐related behaviours [34, 35].

The clinicians and institutions involved in the evolution of disease definitions have demonstrated limited awareness of potential harms to the individual and the healthcare systems [32]. It is thus crucial that these changes in disease definition constitute an evidence‐driven process supported by clear benefits to the patients.

## Broadening disease or risk factor definitions

More than 30 years ago, Geoffrey Rose demonstrated the weaknesses of a prevention strategy focused mainly on treating high‐risk individuals. Since globally only small numbers of events occur among individuals at the highest risk in a population, this strategy fails to prevent disease in a larger number of cases arising from the intermediate/low‐risk group, where the risk is small but relevant. This notion highlighted the need ‘to control the determinants of incidence, to lower the mean level of risk factors, and to shift the whole distribution of exposure in a favourable direction’ [36]. The problem is the definitions of baseline levels for intervention.

In modern medicine, several conditions are defined not by the presence of symptoms or any clear pathological sign, but instead by the possibility of developing a specific disease in the future [37]. Many patients with hypertension, high cholesterol or diabetes have no detectable symptoms but are at an increased risk of symptomatic disease. Labelling of risk factors as diseases also implies that the risk of future disease is modifiable by an available and safe intervention [37].

Despite the need to target and treat the numerous cases emerging from lower risk strata, Rose did not argue for changing the diagnostic threshold for treating disease [38]. But, as discussed earlier, we are labelling more and more of the population as ‘diseased’, while trying to detect and intervene early, hoping to prevent more severe disease or future complications. However, and as already stated, widening disease definitions cause harm by ‘exposing more patients to the adverse effects of treatments, triggering an investigation and prescribing cascades, increasing anxiety, and placing a financial burden on patients and the wider society’ [39].

Reviewing disease definitions is normally a purview of specialist guideline groups that tend to expand them by lowering thresholds of normal diagnostic ranges (with the inclusion of more low‐risk patients), creating prediseases (prehypertension, prediabetes), inducing overmedicalization of common or mild life experiences or by changing diagnostic processes [40].

The key issue here is to unveil the complex relationship between the benefits and harms of this disease labelling and the interventions they trigger. As Chiolero et al. [38] note, the relationship between a risk factor and the absolute risk of disease is not linear, meaning that, for an identical absolute reduction, the resulting benefit will be much smaller for individuals within the low‐risk strata than within the high‐risk strata. But while the magnitude of the benefit depends on the baseline risk, the probability of harm is generally constant for each intervention. As so, in patients with earlier or milder disease, it is quite difficult to evaluate the net benefit, and individuals are more likely to be harmed [32, 41].

When considering this balance, one should also understand that although risk can be objectively measured, the level we accept as tolerable is defined arbitrarily. Thus, understanding overdiagnosis due to changing disease definition, and its consequences, can be challenging.

The first clear consequence of broadening the definition of a disease is a distorted perception of trends in prevalence. Including more patients in a diagnosis will certainly increase the prevalence of this disease (Table 2). Nevertheless, this does not reflect a true spike, but only a direct effect of new, larger diagnostic boundaries [7].

**Table 2:** Influence of disease definitions in prevalence of disease

Disease/risk factor	What has changed?	What was the consequence?
Autism	Definition, including more features and subpopulations	Increase in prevalence by 176%
Thyroid cancer	Improvement in diagnostic tools	2.4‐Fold increase of prevalence in the United States; no difference in mortality
	Screening program in South Korea from 1999 to 2008	15‐Fold increase in incidence of papillary thyroid cancer; no difference in mortality
Osteoporosis	Definition on National Osteoporosis Foundation (USA) 2008 guideline	Increase in prevalence from 21% to 72%
Myocardial infarction	Definition on European Society of Cardiology/American College of Cardiology 2000 criteria	Increase in prevalence from 18% to 29%
Prediabetes	Definition with lowering cut‐off levels for fasting glucose or glycated haemoglobin	Increase in prevalence from 26% to 50% in China; increase in prevalence from 26% to 31% in the United States
Breast and prostate cancer	Screening programs worldwide	Increase in prevalence overall; no difference in mortality
Chronic obstructive pulmonary disease	Definition solely based on forced expiratory volume in 1 second/FVC ratio on GOLD guidelines	Twofold increase in prevalence in England and Wales; apparent increase in cardiovascular mortality of patients diagnosed by the new criteria but not by the old one

Abbreviation: FVC, forced vital capacity.

One example is the diagnosis of prediabetes by the American Diabetes Association in 2010. By lowering cut‐off levels for fasting glucose or glycated haemoglobin concentrations, the prevalence of prediabetes increased dramatically. For example, this new definition would result in over half of Chinese adults having prediabetes [42].

The logic of creating a new diagnostic category as a predisease is to obtain benefit by precisely identifying those who are at risk of developing a disease, implementing early and effective preventive interventions to change the natural course of disease [42].

However, the evidence does not support this logic, as numerous prospective studies show that more than half of people diagnosed with prediabetes will never evolve to diabetes in their life [15, 43]. This means that applying these thresholds may unnecessarily burden patients, in exchange for limited benefit. Another striking example (already mentioned) is the threefold increase in thyroid cancer incidence [33]. The same scenario occurred with active screening of breast and prostate cancers. In other words, the prevalence of these cancers has increased at the expense of diagnosing clinically irrelevant tumours, as seen by the unchanged global mortality rate [9].

Another example is the GOLD definition of chronic obstructive pulmonary disease (COPD). Previously, COPD was defined using lower limits of normal (LLN) of the ratio of forced expiratory volume in 1 second (FEV1) divided by the forced vital capacity (FVC). These criteria took into account height, age, sex and ethnicity and used 90% confidence intervals of the normal distribution obtained from the data of many thousands of healthy people. In the 1990s, GOLD defined airways obstruction as a postbronchodilator FEV1/FVC of <0.7 with no account of other factors, without providing evidence of the effectiveness of this fixed ratio of airflow obstruction. This controversial definition doubled the prevalence of COPD in England and Wales when compared with the old criteria [44]. Some studies have shown that the GOLD definition leads to significant overdiagnosis. Additionally, patients diagnosed by the GOLD criteria but not by the LLN criteria have an apparent higher prevalence of cardiovascular mortality. This may be the result of misdiagnosing cardiovascular disease as mild COPD using the GOLD criteria [44].

As said, another direct consequence of changing the definition of a disease is a distorted perception of the success of treatment. These new definitions tend to include patients with milder or earlier disease (or even only an increased estimated risk), who will be less likely to suffer severe consequences but will still be considered in the total number of cases diagnosed. Consequently, health outcomes will appear to improve, even if there is no actual effect. This underlines the importance of understanding the natural history of those diagnosed by the new definition. These additional patients may have a considerably different disease with a better prognosis, improving the average prognosis of all patients classified by the new definition. Randomised trials with a no‐treatment or standard treatment arm can give information about both the ability of a certain disease definition to predict clinically important events and the response to treatment made upon this redefinition. However, one must be cautious about the generalizability of the results, given the frequently strict inclusion/exclusion criteria in these trials. We have mentioned some examples that exactly show this false improvement in outcomes, resulting in a change in disease definition. Concerning the diagnosis of prediabetes, we have discussed how many of the patients labelled with this condition may never have a future diagnosis of diabetes, even without treatment. If one analyses the outcomes of treatment for diabetes—considering these prediabetic patients who are now preventively medicated—these will certainly be improved [32, 42].

Cancer screening programs are another example of this problem due to length‐time bias. This bias occurs because screening tests are individual assessments in each period of time. Consequently, these tests are more likely to detect slow‐growing cancers, which are present longer without symptoms. Fast‐growing cancers will more likely be symptomatic before we have an opportunity to screen for them. Slow‐growing tumours have an intrinsically better prognosis, improving mortality rates at the expense of less severe cases.

**Fig. 3:**
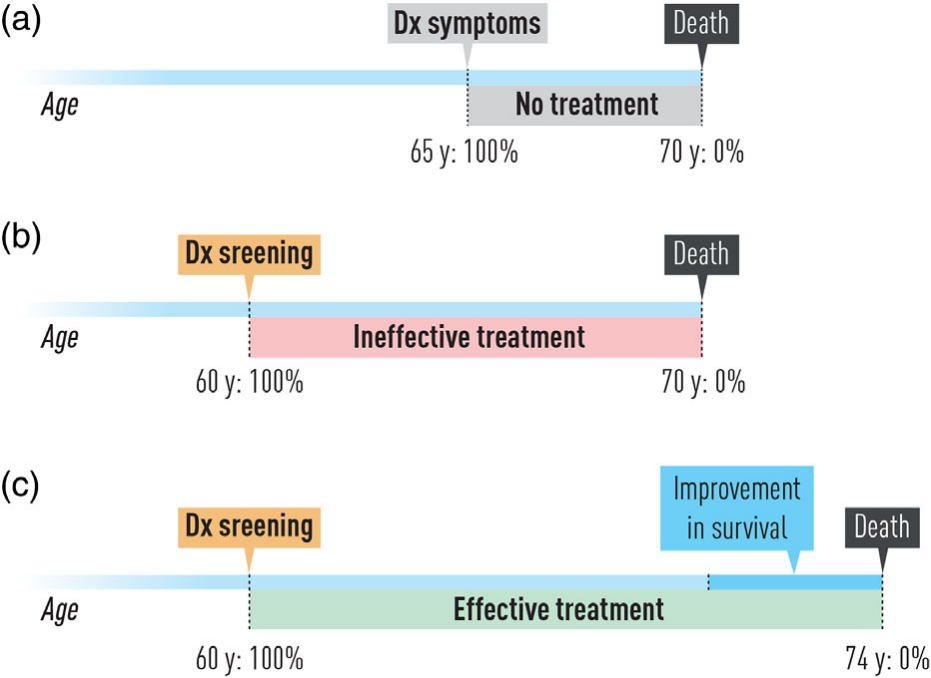
Lead time affects survival time after screening.

Another important bias in cancer screening is lead‐time bias. This bias occurs when a screening test detects a tumour at an earlier time point than it would have been if it had been diagnosed by its clinical picture (Fig. 3). People who are screened appear to survive longer from the time of diagnosis than people who are diagnosed when symptomatic, even if early treatment is not more effective than treatment at the time of clinical manifestations. In other words, the survival time becomes longer only because we are adding the lead time to the normal time from symptoms to death. This creates a bias, as we are only improving the ‘disease time’—the time we acknowledge the patient has a disease—and not the actual ‘survival time’ [45].

The redefinitions of disease also have a great impact on healthcare costs and health systems’ sustainability [40]. For example, one study with over 150,000 Chinese patients showed that the change in definition of (again) hypertension, diabetes and hypercholesterolaemia in the 2000s would double the prevalence of each condition in China. If every patient were medicated, that would have consumed 56% of the total health expenditure in 2010 [34]. Furthermore, these disease definitions with questionable clinical value may lead to a diversion of healthcare resources and attention to treat those with mild disease, threatening the sustainability of healthcare systems worldwide. This results not only in overtreating some patients but also in undertreating others.

Another example is the renaming of chronic kidney disease. KDIGO definition of chronic kidney disease includes around 50% of older people, even if many will never experience any related problems. Reduced levels of kidney function are physiologically normal in older age groups. Renaming some stages of renal impairment as categories of ‘kidney ageing’ could improve patients' understanding of the real importance of their condition and its implication for their health [40, 46].

Finally, how should we detect overdiagnosis in clinical practice? Glasziou et al. advise the use of ‘red flags’ and questions to identify this problem [47].

## Conclusions

After discussing the impact of overdiagnosis and overtreatment in numerous contexts, it becomes clear that we need a paradigm shift in how diseases are defined. What could be done to address this problem?

Undoubtedly, the first step would be estimating the overdiagnosis dimension across common conditions and the respective harm and waste. Research on tests and treatments should aim to better identify treatment thresholds where benefits are likely to outweigh harms and consider most medical conditions as a spectrum of severity rather than simple dichotomous identities [33, 40]. For example, when assessing a new diagnostic test, there should be an evaluation of its capacity of detecting clinically meaningful abnormalities, rather than only its sensitivity and specificity and predicted values. The same applies to treatment, where the approach should focus, wherever possible, on meaningful outcomes that matter to people rather than a blind improvement on real or surrogate markers [33].

Second, there is a need to rigorously evaluate and challenge modifications to disease definitions. This implies a reformed process of reclassifying diseases, driven by multidisciplinary teams without financial conflicts of interest. We believe primary care specialists can have a unique role here since disease specialists tend to focus on preventing serious conditions they often treat in their practice, overtreating patients at lower risk [40]. These factors grant the general practice groups a unique sense of priorities and the balance between harm and benefit. Additionally, this process should meticulously answer a checklist, such as GIN Overdiagnosis Working Group [32]. This checklist outlines questions to be explicitly answered before proposing changes to definitions, such as the number of people who will be affected by the new definition, potential benefits for the newly diagnosed and harms.

Third, following the much‐needed research on the impact of overdiagnosis mentioned above, there should be a shift to a culture of different diagnosing, dediagnosing, deprescribing and de‐implementation of inappropriate health interventions, especially if controversial. On the one hand, similar to a practice of medication review, a frequent diagnostic review in primary care could reduce unneeded labels and treatments. On the other hand, de‐implementation may involve removing, replacing, reducing or restricting the delivery of an ineffective, untested intervention or one which has contradicted or mixed data. This would improve medical practice, establishing diagnoses only when it is believed they will bring more benefit than harm. Another interesting paradigm change would be to discuss the need for a specific diagnosis and treatment with the patient when there is controversy and uncertainty among the medical community. We believe this to be a more flexible and patient‐centred approach, helping maintain public trust, minimize patient harm and reduce unnecessary costs [40, 48].

Finally, there should be a rise in awareness of how diseases are redefined and their inestimable consequences—something this article also aims to do. Debates during medical school, international meetings dedicated to this subject, journal sections such as JAMA Internal Medicine's ‘Less is More’ and initiatives like Choosing Wisely and Cochrane Sustainable Healthcare group (a group focused on addressing medical excess) will all lead to an increase in awareness of this type of low‐value healthcare, and the true harm of overdiagnosing and overtreating patients.

## Conflict of interests

The authors declare no competing interests.

## Author contributions

António Vaz Carneiro: conceptualization; methodology; supervision. All authors contributed equally to the text.
